# The excessive response: a preparation for harder conditions

**DOI:** 10.1007/s13238-017-0454-y

**Published:** 2017-08-01

**Authors:** Yaguang Ren, Chao Zhang

**Affiliations:** Translational Medical Center for Stem Cell Therapy and Institute for Regenerative Medicine, Shanghai East Hospital, Shanghai Key Laboratory of Signaling and Disease Research, School of Life Sciences and Technology, Tongji University, Shanghai, 200433 China

Reactive oxygen species (ROS) are kinds of reactive chemicals mainly formed in mitochondria as byproduct of normal metabolism of oxygen (Balaban et al., 2005). Because of the high reactiveness they usually react with proteins, lipid, and nuclear acids (Labuschagne and Brenkman, 2013; Landolfo et al., 2008), and in theory may participate in every aspects of cellular metabolism. The effects of ROS can either be good or bad for organisms. For example, they participate in cell respiration as intermediate products (Devasagayam et al., 2004), act as signals in glucose stimulated insulin secretion (Pi et al., 2007), and be used to attack pathogens in plants (Allan and Fluhr, 1997; Zeng et al., 2015) and microbes in mammals (Kim et al., 2013). However, they will cause damages including protein carbolynation, lipid peroxidation, and DNA mutations at high levels, which contribute to disruptions of cellular homeostasis (Dan Dunn et al., 2015). ROS are believed to have correlations with aging, degenerative disorders, and cancer (Devasagayam et al., 2004). There is complicated antioxidant system composed of enzymes and metabolites in organisms. In response to oxidative stresses antioxidant enzymes such as superoxide dismutases, catalases, and peroxiredoxins are activated to help clearing up ROS (Devasagayam et al., 2004). The FOXO/DAF-16 and Nrf2/SKN-1 mediated redox pathways are also reported to be involved in those processes in mammals and the nematode *C*. *elegans* (Pi et al., 2007; Putker et al., 2013; Staab et al., 2013). How does the antioxidant system in organisms respond to high level of prooxidant stresses? Recent study shown in *C*. *elegans* that the response was not only sufficient but also excessive (Ren et al., 2017). When prooxidant stress goes high the antioxidant capacity goes higher and lower levels of reactive oxygen species (ROS) will be observed (Ren et al., 2017). Just like throwing a ball onto the ground: the stronger the force the higher the height will be.

**Figure 1:**
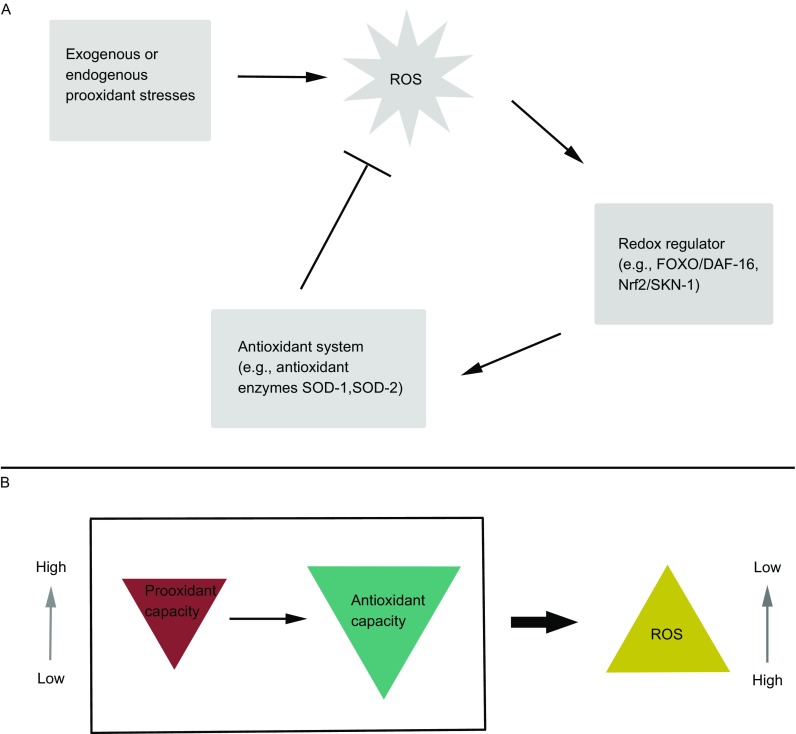
**Description of the excessive response of the antioxidant system under prooxidant stresses**. (A) Exogenous or endogenous prooxidant stresses promote the generation of ROS. The antioxidant system is persistently and excessively activated by the redox regulators and ROS generation is inhibited in the long term. (B) At the level beyond some threshold, when the prooxidant capacity goes high the antioxidant capacity will go higher and lower ROS levels will be observed

Paraquat (PQ, methyl viologen) is known as the herbicide and is often used as ROS inducer in biomedical studies (Castello et al., 2007; Kielar et al., 2012). We found that ROS increased in worms treated with 0.1 mmol/L or higher concentrations of paraquat for only thirty minutes and there seemed to be a positive correlation between ROS and prooxidant stresses (Ren et al., 2017). However, the correlation between the two changed from positive to negative under prolonged treatments when paraquat concentration was at the range of 0.1 to 0.5 mmol/L. At these levels worms’ growth was retarded and reproduction was reduced although survival was not obviously affected, suggesting sub-lethal effects on worms (Ren et al., 2017). But how could elevated prooxidant stresses lead to lower ROS levels? We believe that although the result is counter intuitive but can be reasonably explained by the “excessive response” of the antioxidant system. In organisms ROS levels should be determined by both the prooxidant and antioxidant capacities. However, when prooxidant capacity elevates to the level beyond some threshold antioxidant mechanisms are excessively activated and lower ROS levels will be observed. That is why ROS increased in worms treated with paraquat for only thirty minutes, because more time should be required for the transcription, translation, and maturation of antioxidant enzymes. This model is also supported by the fact that worms grown on plates containing higher concentrations of paraquat generally showed stronger resistance to adverse conditions (e.g., high salts, heat shock, and extreme oxidative stress) (Ren et al., 2017), and transcription of antioxidants and chaperones were increased under oxidative stresses (Ren et al., 2017; Shin et al., 2011; Zarse et al., 2012). In addition, mutations of *sod2*/*3* or the redox regulator *daf-16* abolished the negative correlation between ROS and paraquat levels, suggesting major effects of the excessive response was contributed by the antioxidant system (Ren et al., 2017). Besides FOXO/DAF-16, the Nrf-2/SKN-1 factor was also reported to participate in the antioxidant response (Staab et al., 2013). Our findings are consistent with previous study which showed that increased mitochondrial metabolism and ROS levels due to increased respiration activated antioxidant enzymes and led to further decrease of ROS in the long term (Zarse et al., 2012). Based on the combined results we here proposed the “excessive response” concept illustrated in Fig. 1. It should be noted that persistent prooxidant stresses may exhaust worms by keeping the prooxidant and antioxidant capacities both at high levels and are thus detrimental. However, we do not exclude the possibility that discontinuous prooxidant treatments may still be beneficial and further efforts are required to investigate into this topic.

The excessive response may be a preparation for unpredictable and harder conditions forthcoming. If the extent of the response is only appropriate but not excessive, the organism will be killed immediately by worse environmental factors before it has time to motivate stronger protective mechanisms. Such kind of response may also exist in other cellular processes besides ROS metabolism. It is well known that physical activity is beneficial for health in terms of prevention of heart disease and cancer, fat control, and maintenance of mental function (Berra et al., 1977; Douchi et al., 2000; Hotting and Roder, 2013). However, toxic lactate and ROS are generated during exercise. The paradox can be reconciled by the excessive response which implicates that lactate or ROS will activate stronger protective mechanisms and may protect against potential diseases inducing factors in the long term. Ionizing radiation and ROS inducing strategies are widely used for cancer treatments (Gupta et al., 2012; Kong et al., 2000; Schumacker, 2006). According to the excessive response model survived tumor cells may obtain higher capability to deal with additional ROS-generating insults and higher dose of irradiation, which makes them harder to be killed by the immune system. Thus, “super” tumor cell is born out of adverse environment, which to some extent explains why people with cancer usually die sooner after radiotherapy. Consistently, adaptation to hydrogen peroxide enhances PC12 cell tolerance against oxidative damage (Chen et al., 2005), and worms grown on plates containing higher level of the prooxidant paraquat show stronger resistance to multiple kinds of stresses (Ren et al., 2017). Similar kinds of excessive responses may also exist in lipid metabolism, inflammation, and other cellular processes, which deserve further investigations.
